# Supplementary material for: Big data analyses reveal patterns and drivers of the movements of southern elephant seals
Source: Sci Rep. 2017 Mar 8;7:112. doi: 10.1038/s41598-017-00165-0 (PMC5427936; doi:10.1038/s41598-017-00165-0)
Supplement: Supplementary file 1 — Supplementary Information [file 41598_2017_165_MOESM1_ESM.pdf]

Big data analyses reveal patterns and drivers of the movements of southern elephant seals

Jorge P. Rodríguez, Juan Fernández-Gracia, Michele Thums, Mark A. Hindell, Ana M. M. Sequeira, Mark G. Meekan, Daniel P. Costa, Christophe Guinet, Robert G. Harcourt, Clive R. McMahon, Monica Muelbert, Carlos M. Duarte, Víctor M. Eguíluz

Supplementary Information (SI)

SI Figure legends

Figure S1. Observation time series for each individual. Different colours correspond to elephant seals tagged at seven different deployment locations: Macquarie Island (blue), Campbell Island (orange), Kerguelen Island (yellow), Casey station (red), Davis station (green), Livingston Island (magenta), and Elephant Island (cyan).

Figure S2. Step length analysis of the trajectories. **a**, Step length probability density function; **b**, Step length for different temporal scaling λ[1]. The steps have been normalized dividing by the average step length for every temporal rescaling. Turning points are obtained after projection along the latitude axis[2]. The pdf shows a double power-law scaling. The tail of the pdf shows a scaling domain that shrinks as the rescaling parameter increases and whose exponent depends also on the rescaling parameter decreasing from a value close to 2. For intermediate step lengths the pdf shows a scaling compatible with a power law with exponent 0.6; **c**, Angle distribution for consecutive displacements for two different time windows, 0.5 days (red) and 10 day (black).

Figure S3. Mean displacement (black) and root mean square displacement (red) scaling with time *T*. Both the mean displacement (black), $\langle d(T) \rangle$, and the root mean square displacement (red), $\sqrt{\langle d^2(T) \rangle}$, show the same scaling with *T*: T^{α}, with α=0.83.

Figure S4. Spatial density of observations. Probability density function of the number of observations per grid cell area ρ_{ev}.

Figure S5. Occupancy map of the reshuffled trajectories. Inset: comparison of the trajectories representing three realizations of the reshuffled (red, green and blue) and observed (black) locations of one individual. The colour scale is logarithmic. Figure generated with Matplotlib Basemap Toolkit[3].

Figure S6. Diversity of trajectories according to their normalized entropy *S*. Probability density function of the entropy of all the trajectories.

SI Figures

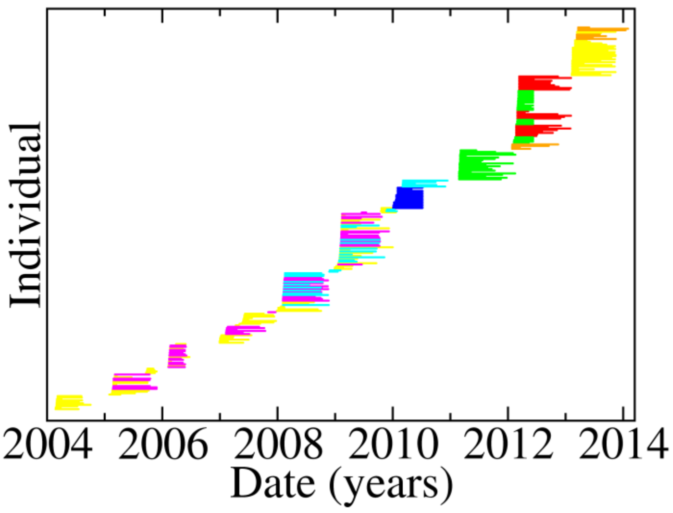

Figure S1.

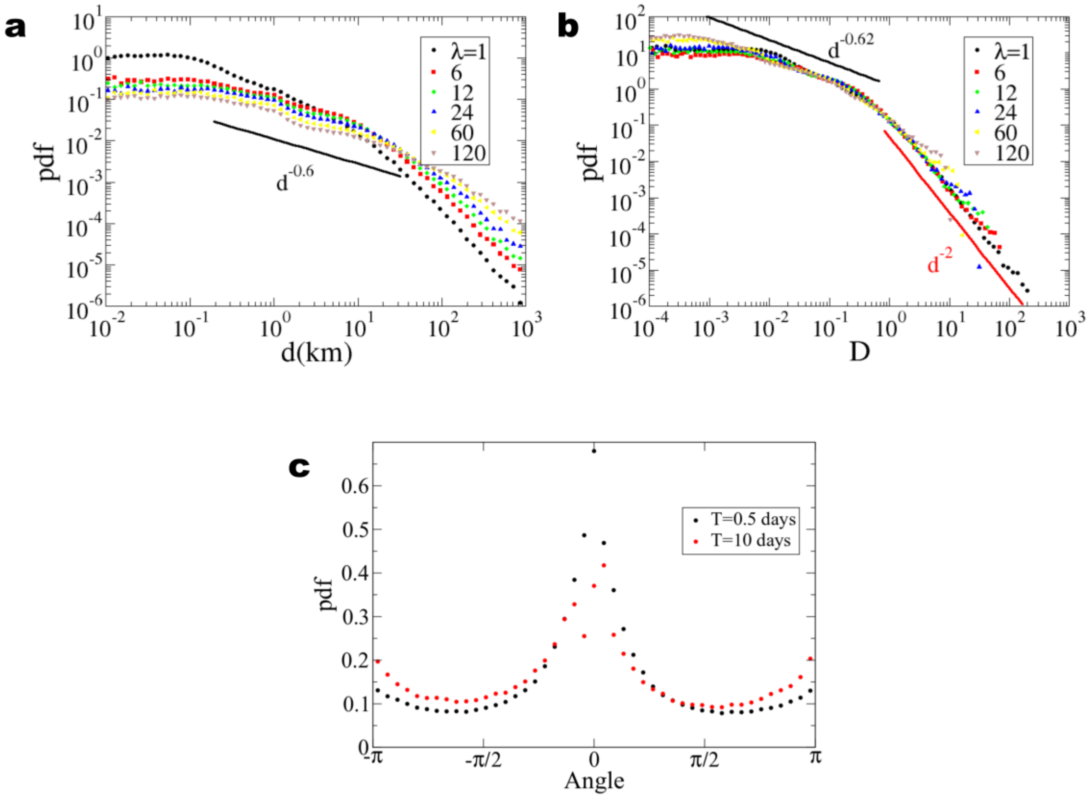
a
pdf
d(km)
$d^{-0.6}$
λ=1
6
12
24
60
120
b
pdf
D
$d^{-0.62}$
d^{-2}
λ=1
6
12
24
60
120
c
pdf
Angle
T=0.5 days
T=10 days
-π
-π/2
0
π/2
π

Figure S2.

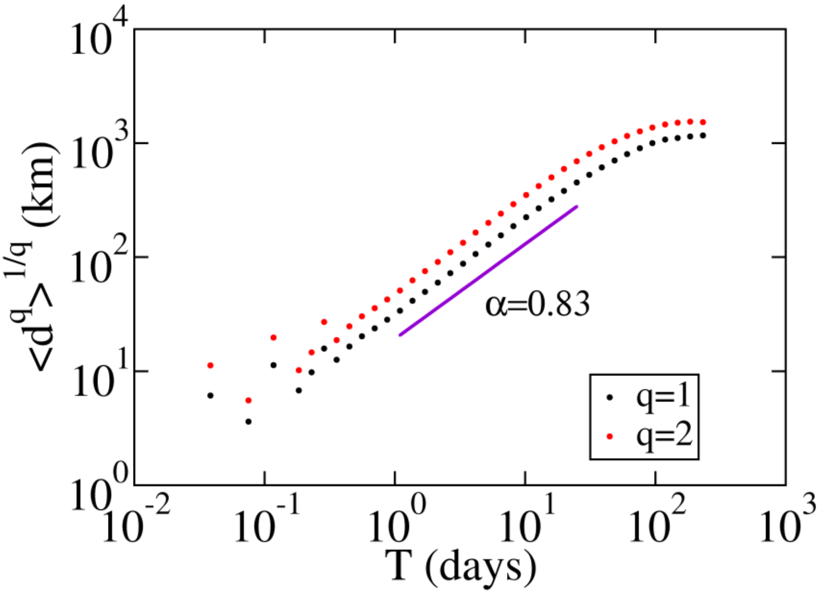

10^4
10^3
10^2
10^1
10^0
$<d^q>^{1/q}$ (km)
10^{-2}
10^{-1}
10^0
10^1
10^2
10^3
T (days)
α=0.83
q=1
q=2

Figure S3.

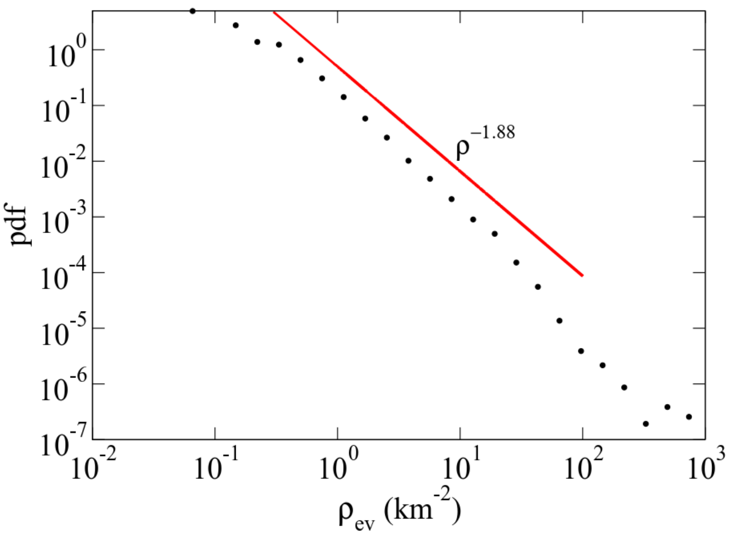
pdf
10^{0}
10^{-1}
10^{-2}
10^{-3}
10^{-4}
10^{-5}
10^{-6}
10^{-7}
10^{-2}
10^{-1}
10^{0}
10^{1}
10^{2}
10^{3}
$\rho^{-1.88}$
ρ_{ev} (km^{-2})

Figure S4.

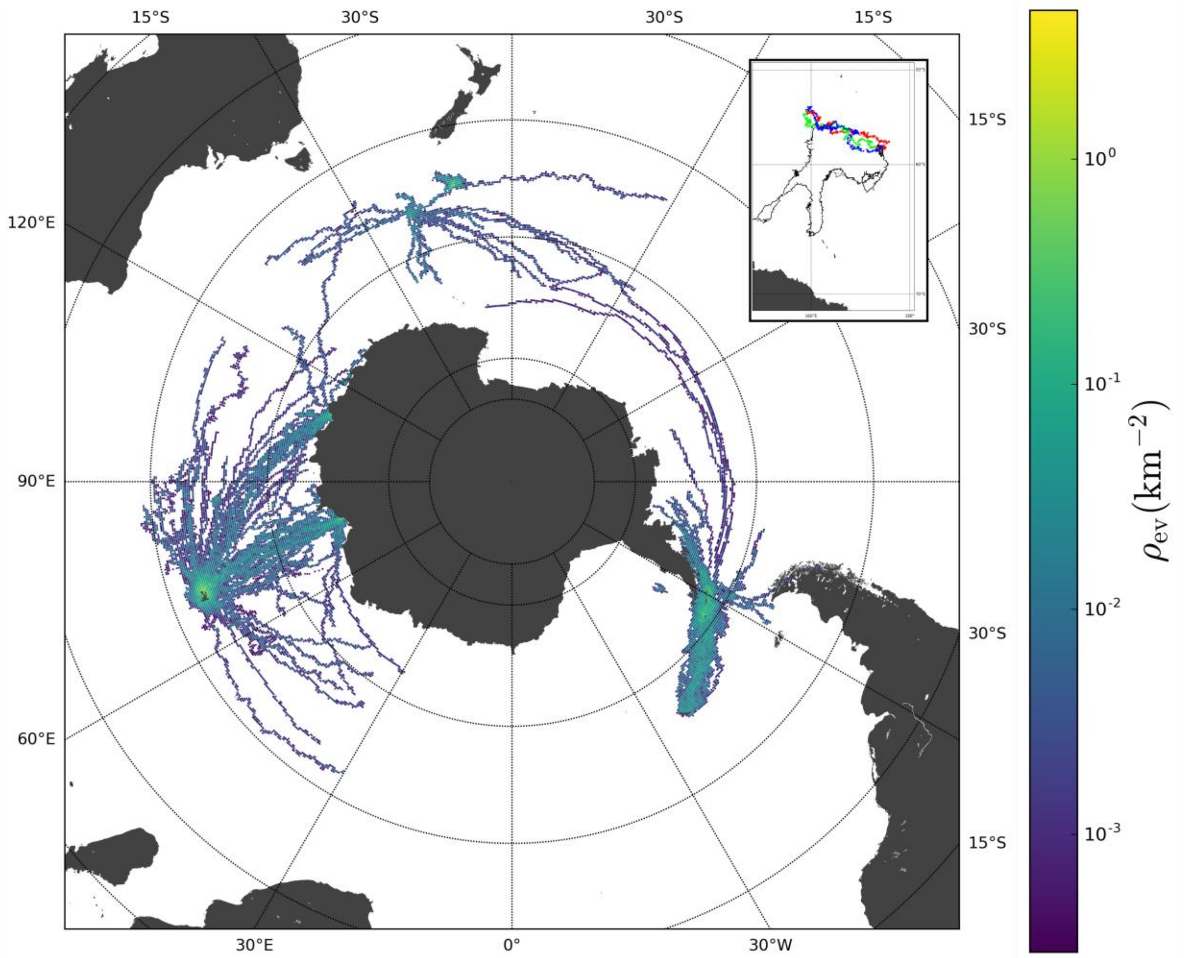
15°S
30°S
30°S
15°S
15°S
120°E
30°S
90°E
30°S
60°E
15°S
30°E
0°
30°W
10^0
10^{-1}
10^{-2}
10^{-3}
$\rho_{\mathrm{ev}}(\mathrm{km}^{-2})$

Figure S5.

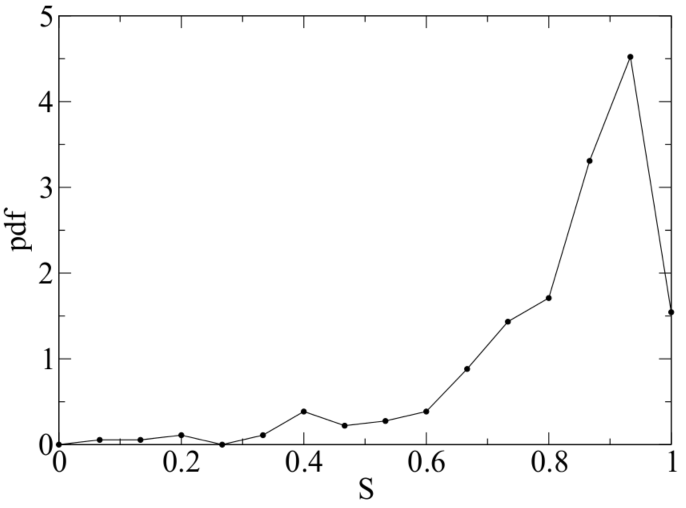

Figure S6.

References

1 Tromer, R. *et al.* Inferring Lévy walks from curved trajectories: A rescaling method. *Physical Review E* **92**, 022147 (2015).

2 Humphries, N. E., Weimerskirch, H. & Sims, D. W. A new approach for objective identification of turns and steps in organism movement data relevant to random walk modelling. *Methods in Ecology and Evolution* **4**, 930-938 (2013).

3 Hunter, J. D. Matplotlib: A 2D graphics environment. *Computing in science and engineering* **9**, 90-95 (2007).
